# Tobacco use and nicotine dependence among people living with HIV who drink heavily in South Africa: a cross-sectional baseline study

**DOI:** 10.1186/s12889-019-8047-8

**Published:** 2019-12-16

**Authors:** Catherine O. Egbe, Mukhethwa Londani, Charles D. H. Parry, Bronwyn Myers, Paul A. Shuper, Sebenzile Nkosi, Neo K. Morojele

**Affiliations:** 1 Alcohol, Tobacco and Other Drug Research Unit, South African Medical Research Council, No. 1 Soutpansberg Road, Pretoria, 0001 South Africa; 2 Department of Public Health, Sefako Makgatho Health Sciences University, Pretoria, South Africa; 3 Alcohol, Tobacco and Other Drug Research Unit, South African Medical Research Council, Cape Town, South Africa; 4 Department of Psychiatry, Stellenbosch University, Stellenbosch, South Africa; 5 Division of Addiction Psychiatry, Department of Psychiatry and Mental Health, University of Cape Town, Cape Town, South Africa; 6 Institute for Mental Health Policy Research, Centre for Addiction and Mental Health, Toronto, Canada; 7 Dalla Lana School of Public Health, University of Toronto, Toronto, Canada; 8 Department of Psychology, University of Johannesburg, Johannesburg, South Africa

**Keywords:** People living with HIV, Tobacco use, Heavy drinkers, South Africa

## Abstract

**Background:**

People living with HIV (PLWH) who drink alcohol and use tobacco are particularly vulnerable to tobacco-induced diseases due to an already compromised immune system. This study investigated the prevalence and factors associated with tobacco use (cigarette and snuff) among PLWH who drink heavily.

**Methods:**

Participants (*n* = 623) on antiretroviral therapy for HIV who reported heavy drinking using the Alcohol Use Disorders Identification Test (AUDIT) and AUDIT-C were recruited from six hospitals in Gauteng Province, South Africa. The Fagerström test was used to assess nicotine dependence. Chi Square tests and modified Poisson regression analyses were conducted to identify factors associated with tobacco use.

**Results:**

Almost half of the participants reported ever smoking (44.0%; CI: 40.1–47.9) and about a quarter reported ever using snuff (25.5%; CI: 22.2–29.1). Current smokers and current snuff users comprised 27.3% (CI: 23.9–30.9) and 19.1% (CI: 16.2–22.3) of all participants respectively. Among current smokers, 37.9% (CI: 30.8–45.3) were moderately/highly dependent on nicotine. Current ‘any tobacco product users’ (ATPU: use cigarettes or snuff) were 45.4% (CI: 41.5–49.3) while 1.0% (CI: 0.4–2.0) currently used cigarettes and snuff. Adjusted regression analyses showed that, compared to males, females were less at risk of being: ever smokers (Relative Risk Ratio [RRR] = 0.33; CI: 0.27–0.41), current smokers (RRR = 0.18; CI: 0.12–0.25), and ATPU (RRR = 0.75; CI: 0.63–0.89) but were more at risk of ever snuff use (RRR = 5.23; CI: 3.31–8.25), or current snuff use (RRR = 26.19; CI: 8.32–82.40) than males. Ever snuff users (RRR = 1.32; CI: 1.03–1.70), current snuff users (RRR = 1.40; CI: 1.03–1.89) and ATPU (RRR = 1.27; CI: 1.07–1.51) were more at risk of reporting significant depressive symptoms. We found no significant associations between smoking status and years on ART and viral load.

**Conclusion:**

There is a high prevalence of cigarette and snuff use among PLWH who drink heavily. Tobacco use cessation interventions tailored specifically for this population and according to their tobacco product of choice are urgently needed given their vulnerability to ill-health.

## Background

Tobacco use is associated with many health conditions including several types of cancer [1]. The average smoker dies 10 years earlier [2] and starts to suffer disability 12 years earlier than the general population [3]. South Africa has the largest HIV epidemic globally with 19% of the global population of people living with HIV (PLWH), 15% of new infections and 11% of global AIDS-related deaths [4]. As of 2016, there were about 7 million PLWH in the country, with 56% having access to antiretroviral therapy (ART) [4].

Globally, PLWH have a higher tobacco use prevalence than the general population [5–8]. However, there is a dearth of data on tobacco use among PLWH in low and middle income countries [8, 9]. A case control study conducted in South Africa involving 279 men living with HIV and co-infected with tuberculosis (TB) reported a 33% smoking prevalence, a lower median CD4 count, and a higher median HIV viral load among smokers [10]. The odds of being diagnosed with TB were three times higher for current smokers and two times higher for former smokers compared to never smokers [10]. Another South African study found the smoking prevalence among a sample of 1210 PLWH to be alarmingly high with 52% of men and 13% of women in the study confirmed as current smokers using urine cotinine and exhaled carbon monoxide tests [5].

PLWH already have a compromised immune system due to their HIV status but this is worsened by the use of tobacco products thus increasing morbidity and mortality rates among this subpopulation [9, 11]. At the onset of the AIDS epidemic and for many years after, Kaposi Sarcoma (KS) was the most prevalent form of cancer among PLWH [1]. With increased access to anti-retroviral therapy (ART), KS has become rare among PLWH (especially in developed countries) [1] while non-AIDS defining cancers have become more common in this population [12]. Highly active ART has been remarkably successful in prolonging survival among PLWH [13]. However, tobacco use-related conditions such as lung and oropharyngeal cancers [12], chronic obstructive pulmonary disease, and cardiovascular disease now account for a growing proportion of morbidity and mortality in this population [13]. A higher prevalence of smoking among PLWH than the general population has led to a higher risk of lung cancer and cardiovascular disease among PLWH [14]. Smoking is also a risk factor for TB which is a common cause of death among PLWH in South Africa [11].

PLWH who are heavy drinkers are at an increased risk of a worsened course of HIV/AIDS and are less likely to adhere to their medication [15, 16]. Alcohol use interacts with the immune system consequently compromising it further [15]. A study of tobacco smoking and alcohol use among PLWH conducted in Nepal found drinking alcohol to be more likely associated with current tobacco smoking among PLWH [17]. A correlation between alcohol and tobacco use has also been found in the general population [18]. High rates of depressive episodes have been reported by PLWH. In a study involving 1187 PLWH in Nigeria, 28.2% of the participants were positively diagnosed with major depressive episodes [19].

Globally, there is a dearth of research on the use of tobacco among PLWH who drink heavily. There are a few studies investigating tobacco use among the general population of PLWH in South Africa and no study has examined tobacco use among PLWH who drink alcohol. The aim of this study is to investigate tobacco use among a sample of PLWH who drink heavily. We examined the demographic characteristics and health outcomes of PLWH who drink heavily and use tobacco, in order to inform targeted and tailored tobacco use cessation interventions for this sub-population. Factors associated with tobacco use were also explored.

## Methods

### Design

The methodology of the larger study has been described in detail elsewhere [20]. This paper reports on the cross-sectional baseline data on tobacco use and nicotine dependence among people who participated in a randomized controlled trial testing the efficacy of an alcohol-focused intervention to reduce alcohol consumption and improve HIV treatment outcomes [20]. Data were collected from May 2016 to October 2017 across six study sites.

### Participants and procedures

Participants for the study were recruited at ART clinics in four district hospitals and two tertiary hospitals in the Tshwane Metropolis, Gauteng Province in South Africa. The province was chosen because it houses the nation’s capital and has the highest population of all provinces, with the most diverse demographic characteristics. The inclusion criteria for the study sites were ART clinics that could guarantee availability of ART for the duration of the project and having a large active caseload of PLWH receiving ART (e.g. at least 100 per week) to facilitate patient recruitment [20]. Participants were included in the study if they were residing in the Tshwane metropolis, 18 years and older, HIV positive, had been on ART for a minimum of 3 months, not being treated for TB, screened positive for heavy alcohol use based on an AUDIT-C score ≥ 4 for males and ≥ 3 for females and a total AUDIT score of < 23, and of good general health. Participants were excluded if they were enrolled in another trial. Questionnaires were administered to the participants by trained fieldworkers in private spaces within the study sites, while qualified phlebotomists collected blood to assess participants’ viral load and recent alcohol consumption. Out of 3054 participants screened, 623 who met the eligibility criteria and consented to participate in the study were enrolled and completed baseline assessments.

### Assessment tools

This paper focuses on data collected on tobacco use, nicotine dependence, demographic and health outcomes (duration on ART, viral load, and depressive symptoms). Demographic questions in the baseline assessment [20] included: age, gender, marital status, education, employment, sources of income, and monthly income. Socioeconomic status (SES) was derived from questions in an asset index. The asset index score was based on ownership of a radio, television, landline telephone, refrigerator, computer/laptop and washing machine; as well as access to electricity. Assets were assessed on the household level. The asset index scores were categorized as: 0–2 = low SES; 3–5 = medium SES and 6–8 = high SES. Participants were also asked to report on their use of tobacco products (cigarette and snuff only, being the two most common tobacco products used in South Africa).

Tobacco use variables were classified as: ever use (ever experimented with smoking cigarettes, even one or two puffs or ever used snuff), current use (ever smoked cigarettes, past 30 days smoking, and having smoked ≥100 sticks of cigarettes in their lifetime, or ever used snuff and past 30 days snuff use) [21], dual tobacco product use (being a current smoker *and* current snuff user) and ‘any tobacco product use’ (ATPU; being a current smoker *or* snuff user).

Nicotine dependence was assessed only for cigarette smokers using the Fagerstrom Test for Nicotine Dependence (FTND) [22]. The FTND is a scale comprising six questions with the total score for the scale categorized as 7–10 (highly dependent); 4–6 (moderately dependent), and 0–3 (minimally dependent) [14]. The FTND scores were recategorized by merging ‘moderately’ and ‘highly dependent’ categories to form a ‘moderately/highly dependent’ category. Viral load (VL) was determined by a laboratory test while years on ARVs were self-reported. VL was categorized as: low VL (0–10,000); moderate VL (10,001-50,000) and high VL (> 50,000). A short version of the Center for Epidemiological Studies Depression measure with 10 questions (CES-D-10) [23] was used to collect data on depressive symptoms. The scale scores were categorized as ≥10 (significant depressive symptoms) and < 10 (non-significant depressive symptoms). The CES-D-10 has been previously validated for use in South Africa [24].

### Data analysis

Stata version 14.0 [25] was used to perform all the analyses. Cross-tabulation and Chi-square tests were used to assess the percentages and gender differences in participants’ tobacco use, nicotine dependence, and report of depressive symptoms. Unadjusted and adjusted modified Poisson regression analyses were performed to assess relationships between demographic characteristics, health outcomes and tobacco indicators). Variables which had categories that were significant (*p ≤* .05) in the unadjusted modified Poisson regression tests were included in the multiple regression models (modified Poisson regression) to identify the factors associated with tobacco use in this population. The *p*-value ≤ .05 was considered statistically significant for all analyses. Only a small proportion of the participants had missing data, and these were excluded on a case by case basis in all analyses (i.e. missing data were excluded for each analysis for all the statistical tests conducted). In Tables 1 and 2, the number of participants included in each analysis is specified.

**Table 1:** Demographics and health outcomes of study participants

Variable	*n*(*N* = 623)	% (CI)
Gender		
Male	265	42.5 (38.7–46.5)
Female	358	57.5 (53.5–61.3)
Age		
18–24	23	3.7 (2.5–5.5)
25–34	118	18.9 (16.1–22.2)
35–44	278	44.6 (40.7–48.6)
45–54	155	24.9 (21.6–28.4)
55–65	45	7.2 (5.4–9.6)
65+	4	0.6 (0.2–1.7)
Socioeconomic status		
Low	36	5.8 (4.2–7.9)
Average	293	47.0 (43.1–51.0)
High	294	47.2 (43.3–51.1)
Marital Status		
Married	173	27.8 (24.4–31.4)
Cohabiting	55	8.8 (6.8–11.3)
Never married	328	52.7 (48.7–56.6)
DIV, SEP and WID	67	10.8 (8.5–13.5)
Employment		
Unemployed	254	42.4 (38.5–46.3)
Employed	359	57.6 (53.7–61.5)
Education		
< Grade 12	383	86.5 (57.6–65.2)
Grade 12	159	25.5 (22.2–29.1)
> Grade 12	81	13.0 (10.6–15.9)
Years on ARV		
Less than one year	27	4.4 (3.0–6.3)
1–3 years	115	18.6 (15.7–21.8)
4–6 years	172	27.8 (24.4–31.5)
7 years and more	305	49.3 (45.3–53.2)
Viral Load		
Low VL	539	92.3 (89.9–94.2)
Moderate VL	25	4.3 (2.9–6.3)
High VL	20	3.4 (2.2–5.3)
Depression		
Non-significant symptoms	334	53.6 (49.7–57.5)
Significant symptoms	289	46.4 (42.5–50.3)

**Table 2:** Tobacco use by gender among PLWH who drink heavily in South Africa

Variable (n)	Male % (CI)	Female % (CI)	Total	p*
Ever smoking (*N* = 623)^a^				< 0.001
Yes (274)	71.3 (65.6–76.5)	23.7 (19.6–28.4)	44.0 (40.1–47.9)	
No (349)	28.7 (23.5–34.4)	76.3 (71.5–80.4)	56.0 (52.1–59.9)	
Age of first try (*N* = 264)^b^				0.152
6–15 years (59)	26.1 (20.2–32.7)	13.2 (7.0–22.1)	22.4 (17.6–27.7)	
16–20 years (121)	44.2 (37.2–51.3)	50.0 (38.9–61.1)	45.8 (39.9–51.9)	
21–25 years (53)	18.1 (13.1–24.1)	25.0 (16.3–35.5)	20.1 (15.6–25.2)	
26–30 years (20)	6.9 (3.9–11.2)	9.2 (4.2–17.2)	7.6 (4.8–11.2)	
31+ years (11)	4.8 (2.4–8.5)	2.6 (0.6–8.2)	4.2 (2.2–7.1)	
Current cigarette smoking (*N* = 623)^a^				< 0.001
Yes (170)	52.1 (46.1–58.0)	8.9 (6.4–12.4)	27.3 (23.9–30.9)	
No (453)	47.9 (42.0–54.0)	91.1 (87.6–93.6)	72.7 (69.1–76.1)	
Nicotine dependence (*N* = 170)^c^				0.096
Minimally dependent (106)	59.1 (50.7–67.1)	75.0 (57.3–87.1)	62.1 (54.7–69.2)	
Moderately/Highly dependent (64)	40.9 (32.9–49.4)	25.0 (13.0–42.7)	37.9 (30.8–45.3)	
Ever snuff use (*N* = 623)^a^				< 0.001
Yes (159)	7.2 (4.5–10.8)	39.1 (34.2–44.2)	25.5 (22.2–29.1)	
No (464)	92.8 (89.3–95.5)	60.9 (55.8–65.8)	74.4 (71.0–77.8)	
Current snuff use (*N* = 623)^a^				< 0.001
Yes (119)	1.1 (0.3–3.0)	32.4 (27.7–37.4)	19.1 (16.2–22.3)	
No (504)	98.9 (97.0–99.7)	67.6 (62.6–72.3)	80.9 (77.7–83.8)	
Dual Tobacco use (*N* = 623)^a^				0.198
Yes (6)	0.4 (0.1–2.6)	1.4 (0.6–3.3)	1.0 (0.4–2.0)	
Any tobacco product Use (*N* = 623)^a^				0.001
Yes (294)	52.8 (46.8–58.8)	39.9 (35.0–45.1)	45.4 (41.5–49.3)	
No (329)	47.2 (41.2–53.2)	60.1 (54.9–65.0)	54.6 (50.7–58.5)	

^a^baseline: overall sample

^b^baseline: ever smokers

^c^baseline: current smokers

*Chi Square test with *p* significant at ≤ .05

## Results

### Demographic characteristics of participants

Just over half of the participants in our study were female (57.5%) (Table 1). The largest group of participants (44.6%) were between 35 and 44 years old. About 94% of the participants reported either medium or high socioeconomic status (SES) in almost equal proportions. Just over half of the participants (52.7%) have never been married and slightly above 40% were unemployed. Only 13.0% of the participants had attained post-secondary education (> Grade 12). Almost half of the participants had been on ARVs for at least 7 years. More than 90% had low viral loads at the time of collecting baseline information. Almost half of all participants (46.4%) reported having significant depressive symptoms.

### Tobacco use among PLWH who drink heavily

The prevalence of ever smoking and ever using snuff in this study was 44.0% (*n* = 274) and 25.5% (*n* = 159), respectively (Table 2). Of participants who had ever smoked, 68.2% (*n* = 180) initiated smoking by the age of 20 while 88.3% (*n* = 233) had tried smoking by the age of 25. Current smokers comprised 27.3% (*n* = 170) and current snuff users comprised 19.1% (*n* = 119) of all participants. One percent of the participants smoked cigarettes and used snuff (dual users) while almost half of the sample (45.4%, *n* = 294) reported either currently smoking cigarettes or using snuff (ATPU). Among current smokers, 37.9% (*n* = 65) were moderately/highly dependent on nicotine according to the FTND.

Chi square tests exploring gender and tobacco use behaviour (Table 2) showed significant differences between males and females with regards to ever smoking, current smoking, ever snuff use, current snuff use and ATPU. No significant gender differences emerged for the age of first use, nicotine dependence, and dual product use (Table 2).

### Relationship between tobacco use, demographic characteristics and depression among PLWH who drink heavily

In unadjusted modified Poisson regression analyses, females showed decreased risk of being ever smokers (RRR = 0.33; CI: 0.27–0.40), current smokers (RRR = 0.17; CI: 0.12–0.24), and ATPU (RRR = 0.76; CI: 0.64–0.90) and increased risk of being ever and current snuff users (RRR = 5.45; CI: 3.47–8.58 and RRR = 28.62; CI: 9.19–89.14 respectively) compared to males (Table 3). Participants aged between 45 and 54 years were two times more at risk of being ATPU (RRR = 2.37; CI: 1.08–5.24) compared to those aged 18–24 years. Participants aged between 55 and 65 years were three times more at risk of being current smokers (RRR = 3.07; CI: 1.01–9.35) and ATPU (RRR = 2.76; CI: 1.23–6.22) compared to those aged 18–24 years. Compared to participants reporting low SES, participants of high SES were less likely to be current smokers (RRR = 0.61; CI: 0.39–0.97). Also, participants who were never married showed less risk of being ever smokers (RRR = 0.79; CI: 0.65–0.96) and more risk of being current snuff users (RRR = 1.65; CI: 1.07–2.54) compared to those who were married.

**Table 3:** Unadjusted modified Poisson regression investigating tobacco use with demographic characteristics and health outcomes^a^

	Ever smoking		Current smoking		Ever snuff use		Current snuff use		ATPU	
	RRR (95% CI)	*p*	RRR (95% CI)	*p*	RRR (95% CI)	*p*	RRR (95% CI)	*p*	RRR (95% CI)	*p*
Gender										
Male^b^	1		1		1		1		1	
Female	0.33 (0.27–0.40)	< 0.001*	0.17 (0.12–0.24)	< 0.001*	5.45 (3.47–8.58)	0.001*	28.62 (9.19–89.14)	< 0.001*	0.76 (0.64–0.90)	0.001*
Age										
18–24^b^	1		1		1		1		1	
25–34	1.27 (0.70–2.30)	0.436	1.95 (0.65–5.86)	0.235	1.07 (0.41–2.82)	0.888	1.56 (0.38–6.32)	0.535	1.68 (0.74–3.78)	0.212
35–44	1.12 (0.62–1.99)	0.708	1.85 (0.63–5.42)	0.264	1.66 (0.67–4.11)	0.278	2.52 (0.66–9.66)	0.177	2.10 (0.96–4.61)	0.064
45–54	1.43 (0.80–2.56)	0.230	2.52 (0.86–7.43)	0.093	1.48 (0.59–3.76)	0.406	2.15 (0.55–8.41)	0.272	2.37 (1.08–5.24)	0.032*
55–65	1.66 (0.90–3.07)	0.105	3.07 (1.01–9.35)	0.049*	1.66 (0.61–4.53)	0.321	2.81 (0.68–11.63)	0.154	2.76 (1.23–6.22)	0.014*
65+	2.16 (0.97–4.78)	0.059	1.92 (0.26–14.17)	0.524	0	–	0	–	1.15 (0.18–7.44)	0.883
SES										
Low^b^	1		1		1		1		1	
Average	1.00 (0.68–1.47)	0.993	0.76 (0.48–1.18)	0.217	1.21 (0.64–2.30)	0.554	1.60 (0.69–3.71)	0.276	0.97 (0.70–1.35)	0.856
High	0.98 (0.67–1.44)	0.917	0.61 (0.39–0.97)	0.036*	1.10 (0.58–2.10)	0.767	1.20 (0.51–2.82)	0.675	0.74 (0.52–1.03)	0.076
Marital Status										
Married^b^	1		1		1		1		1	
Cohabiting	0.93 (0.68–1.27)	0.649	1.26 (0.83–1.92)	0.285	1.19 (0.70–2.03)	0.524	1.23 (0.61–2.50)	0.566	1.22 (0.89–1.68)	0.209
Never married	0.79 (0.65–0.96)	0.020*	0.88 (0.65–1.18)	0.383	1.27 (0.91–1.78)	0.166	1.65 (1.07–2.54)	0.023*	1.11 (0.90–1.38)	0.319
Others	0.82 (0.60–1.13)	0.227	0.88 (0.55–1.41)	0.589	1.33 (0.82–2.14)	0.246	1.68 (0.94–3.03)	0.082	1.11 (0.81–1.52)	0.507
Employment										
Unemployed^b^	1		1		1		1		1	
Employed	1.28 (1.06–1.54)	0.010*	1.50 (0.13–1.98)	0.004*	0.75 (0.57–0.97)	0.031*	0.65 (0.47–0.90)	0.010*	1.04 (0.88–1.24)	0.635
Education										
< Grade 12^b^	1		1		1		1		1	
Grade 12	0. 81 (0.64–1.02)	0.072	0.78 (0.56–1.08)	0.140	0.55 (0.37–0.80)	0.002*	0.50 (0.32–0.80)	0.003*	0.64 (0.51–0.82)	< 0.001*
> Grade 12	1.18 (0.93–1.48)	0.174	0.98 (0.67–1.43)	0.916	0.56 (0.34–0.93)	0.023*	0.47 (0.25–0.89)	0.020*	0.74 (0.55–0.99)	0.041*
Years on ARV										
< one year^b^	1		1		1		1		1	
1–3 years	1.40 (0.84–2.38)	0.199	1.15 (0.61–2.16)	0.677	0.86 (0.39–1.92)	0.714	0.63 (0.27–1.45)	0.275	0.98 (0.63–1.51)	0.911
4–6 years	1.24 (0.74–2.08)	0.416	0.94 (0.50–1.77)	0.852	1.10 (0.52–2.34)	0.806	0.84 (0.39–1.81)	0.652	1.94 (0.62–1.44)	0.782
7+ years	1.08 (0.65–1.80)	0.768	0.80 (0.43–1.48)	0.470	1.31 (0.63–2.72)	0.463	0.96 (0.46–2.01)	0.912	0.92 (0.61–1.39)	0.689
Viral Load										
Low VL^b^	1		1		1		1		1	
Moderate VL	1.09 (0.72–1.65)	0.696	1.49 (0.90–2.45)	0.120	0.61 (0.25–1.51)	0.283	0.60 (0.21–1.77)	0.359	1.14 (0.77–1.68)	0.510
High VL	0.79 (0.43–1.45)	0.452	0.93 (0.43–2.01)	0.852	0.19 (0.03–1.29)	0.089	0	–	0.55 (0.26–1.18)	0.123
Depression										
Non-significant^b^	1		1		1		1		1	
Significant	1.00 (0.84–1.19)	0.987	1.10 (0.85–1.43)	0.455	1.55 (1.18–2.03)	0.002*	1.71 (1.23–2.38)	0.001*	1.29 (1.08–1.53)	0.004*

*ATPU* any tobacco product use, *RRR* Unadjusted Relative Risk Ratio

^a^Modified Poisson regression with DVs: smoking behaviour; IVs: socio-demographic variables, HIV outcome, and depression

^b^reference variable; “other” marital status includes divorced, separated, or widowed

*significant at *p* ≤ .05

Participants who were employed were more at risk of being ever smokers (RRR = 1.28; CI: 1.06–1.54) but were less at risk of being current snuff users (RRR = 0.75; CI: 0.57–0.97) compared to those participants who were unemployed.

Investigation of participants’ highest educational attainment and their tobacco use status showed that PLWH who attained Grade 12 or a post-secondary education (> Grade 12) were less at risk of being ever snuff users (RRR = 0.55, CI: 0.37–0.80; RRR = 0.56, CI: 0.34–0.93), current snuff users (RRR = 0.50, CI: 0.32–0.80; RRR = 0.47, CI: 0.25–0.89) and ATPU (RRR = 0.64, CI: 0.51–0.82; RRR = 0.74, CI: 0.55–0.99) compared to those who attained less than Grade 12 education. However, participants who reported having significant depressive symptoms showed more risk of being ever snuff users (RRR = 1.55; CI: 1.18–2.03), current snuff users (RRR = 1.71; CI: 1.23–2.38) and ATPU (RRR = 1.29; CI: 1.08–1.53) compared to those who reported insignificant depressive symptoms.

### Factors associated with tobacco use among PLWH who drink heavily

Multiple Modified Poisson Regression analyses were conducted to determine the factors associated with tobacco use (Table 4). Adjusted relative risk ratios are reported in this section.

**Table 4:** Adjusted modified Poisson regression investigating factors associated with tobacco use behaviour

Dependent variable	Covariates	RRR	95% CI	*p*
Ever smoking	Gender			
	Male	1		
	Female	0.33	0.27–0.41	< 0.001*
	Marital Status			
	Married	1		
	Cohabiting	0.95	0.73–1.24	0.703
	Never married	0.98	0.82–1.17	0.822
	Other^a^	0.95	0.71–1.28	0.753
	Employment			
	Unemployed	1		
	Employed	1.01	0.85–1.20	0.883
Current smoking	Gender			
	Male	1		
	Female	0.18	0.12–0.25	< 0.001*
	Age			
	18–24	1		
	25–34	1.99	0.67–5.90	0.217
	35–44	1.74	0.60–5.05	0.306
	45–54	1.79	0.62–5.17	0.282
	55–64	2.04	0.69–5.99	0.195
	65+	1.15	0.18–7.16	0.882
	SES			
	Low±	1		
	Average	0.92	0.66–1.29	0.641
	High	0.78	0.55–1.11	0.165
	Employment			
	Unemployed	1		
	Employed	1.02	0.79–1.31	0.911
Ever snuff use	Gender			
	Male	1		
	Female	5.23	3.31–8.25	< 0.001*
	Employment			
	Unemployed	1		
	Employed	1.05	0.81–1.34	0.731
	Education			
	< Grade 12	1		
	Grade 12	0.61	0.42–0.88	0.008*
	> Grade 12	0.61	0.37–0.98	0.043*
	Depression			
	Non-significant symptoms	1		
	Significant symptoms	1.32	1.03–1.70	0.032*
Current snuff use	Gender			
	Male	1		
	Female	26.19	8.32–82.40	< 0.001*
	Marital Status			
	Married	1		
	Cohabiting	1.10	0.60–2.01	0.770
	Never married	1.24	0.83–1.84	0.293
	Other^a^	1.26	0.75–2.11	0.392
	Employment			
	Unemployed	1		
	Employed	1.01	0.75–1.36	0.958
	Education			
	< Grade 12	1		
	Grade 12	0.57	0.37–0.89	0.012*
	> Grade 12	0.51	0.27–0.93	0.029*
	Depression			
	Non-significant symptoms	1		
	Significant symptoms	1.40	1.03–1.89	0.030*
Any tobacco product use	Gender			
	Male	1		
	Female	0.75	0.63–0.89	0.001*
	Age			
	18–24	1		
	25–34	1.64	0.75–3.61	0.217
	35–44	2.01	0.93–4.34	0.074
	45–54	2.05	0.94–4.47	0.070
	55–64	2.25	1.01–5.03	0.047*
	65+	1.07	0.18–6.36	0.943
	Education			
	< Grade 12	1		
	Grade 12	0.68	0.53–0.86	0.001*
	> Grade 12	0.80	0.60–1.07	0.132
	Depression			
	Non-significant symptoms	1		
	Significant symptoms	1.27	1.07–1.51	0.005*

*RRR* Adjusted Relative Risk Ratio based on modified Poisson regression

^a^“other” marital status includes divorced, separated, or widowed

*significant at p ≤ .05

Compared to males, females were less at risk of being ever smokers (RRR = 0.33; CI: 0.27–0.41), current smokers (RRR = 0.18; CI: 0.12–0.25) and ATPU (RRR = 0.75; CI: 0.63–0.89) but more at risk of being ever snuff users (RRR = 5.23; CI: 3.31–8.25) and current snuff users (RRR = 26.19; CI: 8.32–82.40). Compared to participants who had not completed Grade 12, participants who attained Grade 12 or a post-secondary education were less at risk of being ever snuff users (RRR = 0.61; CI: 0.42–0.88 and RRR = 0.61; CI: 0.37–0.98) and current snuff users (RRR = 0.57; CI: 0.37–0.89 and RRR = 0.51; CI: 0.27–0.93). However, only participants who had completed Grade 12 were less at risk of using any tobacco products (RRR = 0.68; CI: 0.53–0.86). Participants who reported significant depressive symptoms were more at risk of being ever and current snuff users and ATPU (RRR = 1.32, CI: 1.03–1.70; RRR = 1.40; CI: 1.03–1.89 and RRR = 1.27, CI: 1.07–1.51 respectively). In terms of age, participants aged between 55 and 64 years were more at risk of reporting any tobacco product use (RRR = 2.25; CI: 1.01–5.03) compared to those aged between 18 and 24 years.

## Discussion

The smoking prevalence found in our study (27%) is higher than the smoking prevalence in the general population of South Africa which is about 22% (among those aged 15+ years) according to the 2016 South African Demographic and Health Survey (SADHS) [26]. In our study, the prevalence of ATPU (45%) is almost twice as high as for the general population (25%) [26], while more than half of the men in our study currently smoke. A study of smoking among 1210 PLWH conducted in Klerksdorp (in the North West Province of South Africa) also found a current smoking prevalence of about 25% based on self-report and 28% based on exhaled carbon monoxide and urine cotinine tests [5]. The self-reported prevalence rate is slightly lower than what we found in our study. This is also the case with the pooled smoking prevalence among HIV positive men in 28 LMICs (24%) by Mdege and colleagues [9]. Since our study focused only on PLWH who are heavy alcohol drinkers, the higher smoking prevalence found in our study is likely to be due to the close association between alcohol use and smoking reported by several studies both in South Africa and globally [5, 17, 18, 27].

As tobacco use carries many health risks including causing various cancers and cardiovascular diseases, the higher prevalence of its use among PLWH who already have a compromised immune system and who are heavy alcohol drinkers is worrisome. As mentioned earlier, the co-use of tobacco and alcohol is common in society [28, 29] and both substances are known to have reciprocal influences on each other [30, 31]. Studies have shown specific negative effects of tobacco use on PLWH including decreased neurocognitive functioning, impaired T-Cells functioning, increased susceptibility to the known effects of smoking, reduced ART adherence and problematic alcohol use [28, 32–34]. Alcohol use in itself is known to affect HIV treatment outcomes and contribute to poor adherence to ART [35–37]. When PLWH use both alcohol and tobacco, this further compromises their immune systems and may result in even worse HIV treatment outcomes than if they only used one substance. The interaction effect of the co-use of alcohol and tobacco is not well researched [38]. However, Pelucchi and colleagues found that the co-use of alcohol and tobacco increases the risk of aerodigestive tract and liver cancers in the general population [38].

Another consequence of the co-use of alcohol and tobacco is the economic implication of using these two addictive substances for the person who is resource-constrained. In this study, participants with high SES were less likely to be current smokers although this association was not significant after controlling for gender, age and employment status. This finding however implies that some smokers in this population will need to use their limited resources to cater for their smoking and alcohol use. Also, low educational attainment (not completing Grade 12) was found to be associated with both ever and current snuff use in this study. There is a high likelihood that persons with low educational qualifications earn lower salaries. The limited availability of funds among such populations would make it even more costly to maintain their tobacco or alcohol use, further straining household finances.

The high prevalence of tobacco use among PLWH who drink heavily in this study also makes it imperative that tobacco use cessation interventions combined with interventions to curb heavy drinking are urgently provided for this population. Specifically, there is need to introduce screening for tobacco and alcohol use as PLWH are initiated onto ART. This screening should be accompanied by targeted tobacco use cessation interventions and education on the harm of tobacco use, including the use of snuff. These interventions are urgently needed for PLWH in South Africa [39]. As we found that most of the participants who smoke cigarettes were minimally dependent on nicotine, there is a high probability of tobacco use interventions being successful in this population.

If this problem does not receive attention, more PLWH may die early of tobacco-related diseases especially if they also drink heavily. A delay in rolling out these interventions could lead to a reversal of the progress already made in the fight against HIV.

The 2012 US Surgeon General’s report highlighted that 99% of adults who are daily smokers had already initiated smoking by the time they were 26 years of age [40]. As such, our findings that almost 70% of PLWH had initiated tobacco use by the age of 20 is not a surprise. Given the early age of initiation, education about the health hazards associated with tobacco use is especially important for PLWH who are adolescents and young adults since most smokers begin before age 18 [41]. Such interventions should include information on the possible effects of using tobacco on HIV treatment outcomes. Interventions like this will help to discourage the initiation of tobacco use by PLWH who have not yet started using tobacco and would encourage those who have already initiated tobacco use to quit.

Snuff use is associated with various types of cancer [42] including head, neck and oral cancers [43–45] and was found to be positively associated with a TB diagnosis among women living with HIV in South Africa [46]. Similar to our findings, a previous study found that females use snuff more than males in the general population [5], while a study among PLWH found an extremely high (49%) prevalence of snuff use among women living with HIV in South Africa [46]. The high prevalence of snuff use found among women in this study also confirms the need for targeted awareness and cessation interventions. While intervention for men who use tobacco could focus more on cigarettes and other combustible tobacco products, those for women who use tobacco should focus more on snuff use and other smokeless tobacco products.

Nicotine dependence was only investigated for cigarette smokers in this study. Future studies should use the FTND for smokeless tobacco users to ascertain nicotine dependence among smokeless tobacco users to better understand nicotine dependence among all tobacco product users.

A Nigerian study of 1187 PLWH found more than a quarter of the participants diagnosed with major depressive episodes based on the Diagnostic and Statistical Manual of Mental Disorders (DSM)-IV diagnostic criteria [19]. In our study, only snuff use was found to be significantly associated with depressive symptoms. The relationship between depression and snuff use found in this study is of concern. There is need to further explore this link in order to understand why more snuff users reported having moderate to high levels of depressive symptoms.

### Limitations

In this study, tobacco use was self-reported which could lead to participants giving socially desirable answers. Also, self-reported tobacco use status was not confirmed using exhaled carbon monoxide or urine cotinine tests. The findings may also not be generalizable to PLWH attending HIV clinics outside of the Tshwane metropolis. Nicotine dependence was only investigated for cigarette smokers in this study, therefore, our understanding of nicotine dependence in this population is limited considering the high prevalence of snuff users found in this study.

## Conclusion

The co-use of alcohol and tobacco is problematic for the general population and even more problematic for the health of PLWH. A better understanding of the tobacco use culture among PLWH is needed to inform interventions targeted for this population. For this population, information on the reasons for initiating tobacco use, when and why tobacco use was initiated, type of tobacco product used, intention to quit, barriers and access to tobacco use cessation interventions is needed [29]. Targeted cessation interventions, including combination therapy involving both pharmacological and psychological interventions, should be explored to help PLWH who use tobacco to quit. In this region, men may benefit from interventions tailored to address cigarette use, whereas women could benefit from interventions that focus on snuff use.

## Data Availability

The datasets used and/or analyzed for this study are available from the corresponding author on reasonable request.

## Abbreviations

AIDS: Acquired immune-deficiency syndrome
ARRR: Adjusted relative risk ratio
ART: Antiretroviral therapy
ARVs: Antiretrovirals
ATPU: Any tobacco product use
AUDIT: Alcohol Use Disorders Identification Test
AUDIT-C: Alcohol Use Disorders Identification Test (3-item version)
CES-D: Center for Epidemiological Studies Depression
CI: Confidence interval
DIV, SEP and WID: Divorced, Separated and Widowed
DSM-IV: Diagnostic and Statistical Manual of Mental Disorders-IV
FTND: Fagerström Test for Nicotine Dependence
HIV: Human immunodeficiency virus
KS: Kaposis sarcoma
LMICs: Low and middle-income countries
PLWH: People living with HIV
SADHS: South African Demographic and Health Survey
SES: Socioeconomics status
TB: Tuberculosis
URRR: Unadjusted relative risk ratio
VL: Viral load
